# The Role of Dysbiosis in Critically Ill Patients With COVID-19 and Acute Respiratory Distress Syndrome

**DOI:** 10.3389/fmed.2021.671714

**Published:** 2021-06-04

**Authors:** Denise Battaglini, Chiara Robba, Andrea Fedele, Sebastian Trancǎ, Samir Giuseppe Sukkar, Vincenzo Di Pilato, Matteo Bassetti, Daniele Roberto Giacobbe, Antonio Vena, Nicolò Patroniti, Lorenzo Ball, Iole Brunetti, Antoni Torres Martí, Patricia Rieken Macedo Rocco, Paolo Pelosi

**Affiliations:** ^1^Anesthesia and Intensive Care, Ospedale Policlinico San Martino, Istituto di Ricovero e Cura a Carattere Scientifico (IRCCS) per l'Oncologia e le Neuroscienze, Genova, Italy; ^2^Department of Medicine, University of Barcelona, Barcelona, Spain; ^3^Department of Surgical Sciences and Integrated Diagnostics (DISC), Università degli Studi di Genova, Genova, Italy; ^4^Department of Anesthesia and Intensive Care II, Clinical Emergency County Hospital of Cluj, Iuliu Hatieganu, University of Medicine and Pharmacy, Cluj-Napoca, Romania; ^5^Anaesthesia and Intensive Care 1, Clinical Emergency County Hospital Cluj-Napoca, Cluj-Napoca, Romania; ^6^Dietetics and Clinical Nutrition Unit, Ospedale Policlinico San Martino, Istituto di Ricovero e Cura a Carattere Scientifico (IRCCS) per l'Oncologia e le Neuroscienze, Genova, Italy; ^7^Clinica Malattie Infettive, Istituto di Ricovero e Cura a Carattere Scientifico (IRCCS) per l'Oncologia e le Neuroscienze, Genova, Italy; ^8^Dipartimento di Scienze della Salute (DISSAL), Università degli Studi di Genova, Genova, Italy; ^9^Division of Animal Experimentation, Department of Pulmonology, Hospital Clinic, Barcelona, Spain; ^10^Centro de Investigacion en Red de Enfermedades Respiratorias (CIBERES), Madrid, Spain; ^11^Institut d'investigacions Biomediques August Pi i Sunyer (IDIBAPS), Barcelona, Spain; ^12^Laboratory of Pulmonary Investigation, Carlos Chagas Filho Institute of Biophysics, Federal University of Rio de Janeiro, Rio de Janeiro, Brazil; ^13^COVID-19-Network, Ministry of Science, Technology, Innovation and Communication, Brasilia, Brazil

**Keywords:** microbiota, dysbiosis, ARDS, critical care, infection, COVID-19

## Abstract

In late December 2019, severe acute respiratory syndrome coronavirus-2 (SARS-CoV-2) quickly spread worldwide, and the syndrome it causes, coronavirus disease 2019 (COVID-19), has reached pandemic proportions. Around 30% of patients with COVID-19 experience severe respiratory distress and are admitted to the intensive care unit for comprehensive critical care. Patients with COVID-19 often present an enhanced immune response with a hyperinflammatory state characterized by a “cytokine storm,” which may reflect changes in the microbiota composition. Moreover, the evolution to acute respiratory distress syndrome (ARDS) may increase the severity of COVID-19 and related dysbiosis. During critical illness, the multitude of therapies administered, including antibiotics, sedatives, analgesics, body position, invasive mechanical ventilation, and nutritional support, may enhance the inflammatory response and alter the balance of patients' microbiota. This status of dysbiosis may lead to hyper vulnerability in patients and an inappropriate response to critical circumstances. In this context, the aim of our narrative review is to provide an overview of possible interaction between patients' microbiota dysbiosis and clinical status of severe COVID-19 with ARDS, taking into consideration the characteristic hyperinflammatory state of this condition, respiratory distress, and provide an overview on possible nutritional strategies for critically ill patients with COVID-19-ARDS.

## Introduction

In late December 2019, a novel coronavirus able to induce an acute respiratory syndrome was identified in Wuhan, China (1). This virus, since named severe acute respiratory syndrome coronavirus-2 (SARS-CoV-2), quickly spread worldwide, and the syndrome it causes, coronavirus disease 2019 (COVID-19), was declared a pandemic by the World Health Organization on March 11, 2020. The standard presentation of COVID-19 includes fever, dry cough, respiratory distress, fatigue, myalgia, dyspnea, headache, and diarrhea (1, 2). Fecal samples collected from patients with COVID-19 remained positive for about 11 days, raising concerns about the possible fecal-oral transmission of the virus and gastrointestinal symptoms (3, 4).

Several patients with COVID-19 are admitted to the intensive care unit (ICU) due to severe respiratory distress, with a clear status of typical or atypical acute respiratory distress syndrome (ARDS), requiring critical care support (2, 5). The rate of patients admitted to ICU depends on the diversity of cares within countries and the pandemic global situation. Indeed, compared with the first wave of the pandemic, during the second wave 50% less of all hospitalized patients with COVID-19 were admitted to the ICU (6). Traditional critical care includes respiratory and cardiovascular support, management of renal, hepatic, infectious, and neurologic status, and nutritional management (2). Patients with severe COVID-19 often experience an enhanced immune response with a hyperinflammatory state characterized by a “cytokine storm” (7), with fever and respiratory distress considered to represent increased dysbiosis. During critical illness, the multitude of therapies administered, including antibiotics, sedatives, analgesics, invasive mechanical ventilation, and nutritional support, may enhance the inflammatory response and impact on the patients' microbiota, leading to dysbiosis. In turn, this status may lead to hyper vulnerability in patients and an inappropriate response to critical circumstances (8).

In this context, the aim of our narrative review is to provide an overview of possible interaction between patients' microbiota dysbiosis and the clinical status of severe COVID-19 with ARDS, taking into consideration the characteristic hyperinflammatory state of this condition, respiratory distress, and provide an overview of possible nutritional strategies for critically ill patients with COVID-19-ARDS.

## Pathophysiology of the Microbiota Gut-Lung Axes

The gut-lung axis is bidirectional, which means that metabolites derived from the gut or lung bacteria can affect each other. Gut microbiota is often altered as early as the first days of ICU admission (9), altering both susceptibility to and severity of infections (10). Mechanisms implied in microbiota-lung-gut-axis alteration in COVID-19 include: (1.1) Direct lung damage (1.2) ACE2 expression; (1.3) gut microbiota as lungs' defense against SARS-CoV-2; and (1.4) immune response.

### Direct Lung Damage in COVID-19

ARDS is a common complication of COVID-19. After binding to angiotensin-converting enzyme-2 receptors (ACE2) and transmembrane protease serine 2 (TMPRSS2), SARS-CoV-2 enters the host cells and causes pneumonia with possible ARDS in the most severe cases. The histologic presentation of severe COVID-19 pneumonia includes a progressive thickening of the alveolar wall with infiltrates of mononuclear cells and macrophages, associated with endotheliitis (11). Edema, fibroblasts, and myofibroblasts thicken the alveolar septa, with interstitial inflammatory cell infiltrates. In the late and organizing stage, the lungs become consolidated, and fibroblasts and myofibroblasts proliferate and migrate, forming granulation tissue and fibrosis by accretion, with possible thickening of interlobular septum and bronchial walls, thus leading to diffuse alveolar damage (DAD) (11). In this state, patients with severe COVID-19 may need to be admitted to the ICU for endotracheal intubation and mechanical ventilation. The evolution to COVID-19-ARDS is characterized by pulmonary edema, hypoxemia and inflammation, which are associated with changes in the lung microbiome (12). The microbiota is defined as the overall community of microbes included in a population (13), and the genetic content of the microbiota is known as the microbiome. In healthy conditions, the composition of the microbiota is generally characterized by high abundance and diversity of microorganisms with preponderance of potentially beneficial species, a condition known as eubiosis (13).

The microbiota is primarily involved in the immune response and host defense against pathogens, as well as in gut maturation, nutrient uptake and metabolism, mucosal barrier function, intestinal motility, and modulation of the enteric nervous system (14). Moreover, mechanical ventilation, decreased bowel transit time, reduced oxygenation, multiple antibiotic usage, sedatives, analgesics, muscle relaxants, gastric protectors, and abnormal nutritional intake may affect the composition of microbiota, which may increase the risk of dysbiosis and inflammation (15–17). Mice treated with broad spectrum or targeted antibiotics impaired their response to systemic and respiratory infections (18). Most prominent among these are gram-negative bacteria (e.g., *Proteobacteria*), which can lead to severe gut-lung dysbiosis (9, 19).

### ACE2 Expression

Once affected the lungs' tissue, COVID-19 may extend to other organs by binding to ACE2 and TMPRSS2 (20, 21), which are broadly expressed in various tissues (22, 23). ACE2 are involved in the regulation of inflammation and microbial community, while regulating the host intestinal metabolism of tryptophan, which plays a key role in the composition of gut microbiota (24–26). Thus, ACE2 expression may alter both the lung and gut microbiomes in certain disease conditions (24–26). In fact, a down-regulation of ACE2 reduces the absorption of tryptophan in the gut, while reducing the secretion of antimicrobial peptides and triggering dysbiosis (27). *Bacterioides dorei* and other bacterial species down-regulate the expression of colonic ACE2, thus supporting the appearance of intestinal symptoms in some COVID-19 patients (28). SARS-CoV-2 infection of the intestinal tract impairs the absorption of nutrients altering the intestinal function and activation of the enteric nervous system, causing gastrointestinal manifestations (29). Recent findings confirmed the role of gut dysbiosis in the induction of ARDS and its importance in possibly determining tissue damage in SARS-CoV-2 infection (16, 30).

### Gut Microbiota as Lungs' Defense Against SARS-CoV-2

The gut microbiota regulates the function of the immune system and cellular homeostasis of both gut and lung tissues due to antimicrobial peptides and metabolites derived from intestinal commensals (18, 31). The enteric nervous system is composed of the myenteric plexuses, which control fluid movement and intestinal motility; and is influenced by the activation pattern recognition receptors (PRRs), especially toll-like receptors (TLRs) which recognize pathogens (32).

SARS-CoV-2 infection may promote intestinal inflammation, epithelial barrier disruption, and decreased production of antimicrobial peptides (AMPs), until developing secondary enteric infections. An increased inflammatory status of the gut induced by SARS-CoV-2 may alter gut permeability causing epithelial leakage, which may enhance bacterial translocation and trigger systemic inflammation and inflammatory response to other organs (22). Additionally, over-expression of fecal calprotectin is implied in gut inflammation in COVID-19 (33). The passage of gut bacteria from the intestinal to the lung tissues is regulated by the ability of gut tight junctions in maintaining epithelial permeability, and intestinal bacteria in preserving the intestinal barrier. Among the proposed mechanisms of alteration, products of commensal bacteria fermentation like butyrate and other short-chain-fatty-acids (SCFAs) are responsible of intestinal barrier function and regulation of tight junctions' permeability (34). Additionally, the alteration in the secretion of mucin by goblet cells can lead to impaired mucus layer, increasing susceptibility to increased gut permeability (35). Dysbiosis results in diminished production of microbial-associated molecular patterns including TLRs and nucleotide oligomerization domain (NOD)-like agonists and microbial metabolites such as SCFAs, thus reducing antibacterial pulmonary immunity (18). Hence, by altering the gut immune homeostasis, respiratory viral superinfection may occur. Gut bacteria were found in lung microbiome, suggesting possible translocation from the intestinal tract to the lungs via the hematic circulation (36). The abundance of gut-associated pathogens in the lungs is increased in non-COVID-19 ARDS patients, but little is known regarding ARDS associated with COVID-19. Patients with ARDS revealed a higher prevalence of *Lechnospiraceae* as a strong predictor of reduced survival (37). Some authors investigated the lung bacterial burden and diversity of patients with non-COVID-19 ARDS, concluding that the lung microbiota is reduced in term of diversity and is increased in terms of bacterial burden (38).

### Immune Response

**Figure 1:**
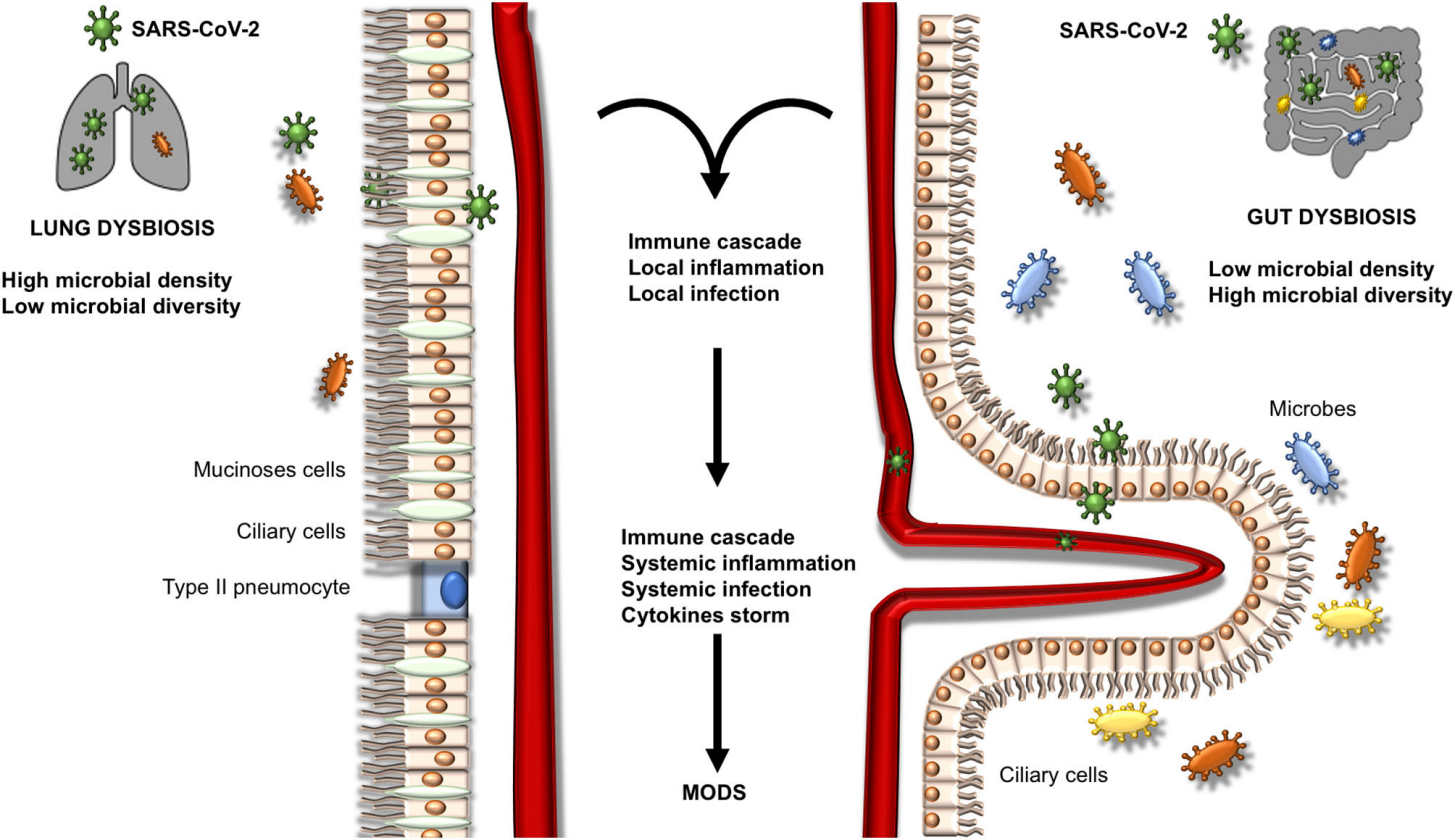
Mechanisms of microbiota gut-lung axis dysbiosis. This figure represents the possible evolution of dysbiosis in the lungs and intestine. A local inflammatory process is activated, thus converting in a systemic inflammatory process with possible infection and multiorgan disease syndrome (MODS).

The multifunctional SARS-CoV-2 Envelope (E) protein, which interact with host tight junction protein ZO1, showed great contribution to virus assembly, while contributing to epithelial barrier damage, pathogenesis (binding to ACE2 receptor), and disease severity (39). Human intestinal epithelial cells (in the esophageal upper epithelia, gland cells, enterocytes of the ileum and colon) are potential target of viral replication, also promoting spreading of SARS-CoV-2 and immune response mediated by type III interferon (IFN) (40, 41). At lungs level, studies have highlighted the impact of gut microbiota on the lungs' production of type I IFN, which is implied in the control of viral infections (42, 43). Microbial metabolites such as desaminotyrosine and SCFAs are critical for microbiota homeostasis. For example, significant changes in the composition of gut microbiota have been identified in an experimental model of pulmonary influenza (44). Desaminotyrosine is produced by an anaerobe clostridium called *Clostridium orbiscindens* to protect the lungs and activate the innate immune response passing through the blood system against influenza infection. Desaminotyrosine implements type I IFN signaling of lungs' phagocytes by promoting genes transcription (45). Similarly, SCFAs result important in priming the pulmonary immune innate system (18, 31). The subsequent inflammatory response can promote and encourage local inflammation followed by endothelial and epithelial injury (Figure 1).

The inflammatory response of SARS-CoV-2 infection is very complex. In fact, SARS-CoV-2 may interfere with the innate antiviral immune response that is made up two different antiviral pathways.

Phagocytes are recruited to fight against local infections and to repair and regenerate the epithelium. As aforementioned, the manipulation of cytokines and IFNs may play an important role in the prevention of infections and mucosal protection. Particularly, IL-22 and IFN-λ act as mucosal defenders and upregulate antimicrobial peptides (46). The IFN regulatory factors increase transcription of type I and III IFNs, which stimulate natural killer cells and cluster differentiation (CD)8+ T lymphocytes, whereas the nuclear factor-kB (NF-kB) promotes the activation of monocytes and their differentiation into macrophages (type M1). Cytokines are therefore released, and T-cells activated (inflammatory T-cells Th1 and Th17). Notably, a “cytokine storm” appears to occur in cases of severe COVID-19, as demonstrated by increased levels of interleukin (IL)-2, IL-17, granulocyte-colony stimulation factor, IFN-γ, inducible protein 10, monocyte chemoattractant protein-1, macrophage inflammatory protein 1-α, and tumor necrosis factor-α (7). Previous studies found that IL-22 is substantially expressed during viral infections, and animals with deficiency of IL-22 were unable to repair and regenerate epithelial tissues (47). Moreover, IL-22 usually enhance the recruitment of other inflammatory cells, thus amplifying the systemic inflammatory response (46, 48), which along with the local damage may predispose COVID-19 patients to secondary bacterial infections, capillary leakage syndrome, and systemic bacterial translocation (49), thus enhancing the risk of multiple organ damage (20–22). Nevertheless, in the COVID-19 era the role of cytokines and interferons on epithelial integrity and systemic reaction is still not clear, and IL-22 and IFN-λ might be considered as further promising targets to maintain the COVID-19 lungs' integrity, but more evidences are urgently needed (50). This exaggerates cytokines and interleukins release may increase the expression of markers like programmed death-1, T-cell immunoglobulin, mucin domain-containing protein-3 while favoring lymphocyte apoptosis and necrosis. Lymphopenia is frequent and is associated with disease severity and inflammation (51). Lymphopenia in COVID-19 may be induced by several mechanisms, including direct infection of lymphocytes, viral aggression of lymphatic organs, or continuous inflammation with cytokines release that could induce lymphocyte deficiency (52–54). Additionally, lymphopenia may be associated with glucocorticoids treatment (51). Since gut microbiota is one of the key components of the host immune system, and primary responses to infections and other immune insults, lymphopenia due to SARS-CoV-2 infection may interfere and predispose to changes in the normal flora by opportunistic germs (52–55).

## Composition of the Gut-Lung Microbiota in Cases of Severe Covid-19 Pneumonia

The gut represents the largest microbial environment in humans. The healthy (eubiotic) intestinal microbiota represents a highly heterogeneous ecosystem including eukaryotic organisms (including *Yeast*)*, Virus, Archaea* and *Bacteria*. The latters are the most represented members and include nine different phyla, of which *Bacteroidetes* and *Firmicutes* represent the most common populations (13, 32). Differently from the gut, the microbiota is scarcely represented in the lung, being mainly associated with mucosal surfaces. The gut microbiota of patients with COVID-19 demonstrated a high prevalence of opportunistic pathogens over commensals that persisted after negative swabs and resolution of respiratory symptoms. The abundance of *Coprobacillus, Clostridium ramosum*, and *Clostridium hathewayi* correlated with the severity of COVID-19 (33). Another study confirmed a high prevalence of opportunistic pathogens in patients with COVID-19, including *Streptococcus, Rothia, Veillonella*, and *Actinomyces* with a reduced relative abundance of symbionts (56).

The high prevalence of gastrointestinal disorders associated with acute infection by SARS-CoV-2 (anorexia, dysgeusia, ageusia, diarrhea, nausea, and hematemesis) (57) might be associated with the damage to the intestinal ecosystem that may be modified (58). In fact, SARS-CoV-2 infection can impact on some tight junction proteins (like PALS1), that compose the intestinal and lung epithelium. However, definitive confirmation on the impact of SARS-CoV-2 on tight junctions and intestinal permeability while potentially damaging to enterocytes are still limited and warrants further molecular researches (59). Gastrointestinal symptoms have been also associated with reduced number of circulating lymphocytes, and the circulating lymphocytes were inversely associated with virus discharge in stool (58). Moreover, a recent meta-analysis on gastrointestinal symptoms of SARS-CoV-2 infection concluded that gastrointestinal symptoms on admission were associated more with complications, including ARDS, acute cardiac injury, and acute kidney injury (57).

The main difference between gut and lung microenvironments is the higher turnover of lung bacteria with regard to the gut counterpart. This characteristic of the lung microbiota is due to the high rate of immigrated and eliminated pathogens (aspiration and mucosal dispersion vs. cough and muco-ciliary clearance). The gut microbiota, which is usually enriched in nutrients, makes tough competition with dense resident communities. In contrast, the lung microbiota is enriched in pharyngeal microbes (60, 61), as demonstrated in numerous studies (62, 63).

**Figure 2:**
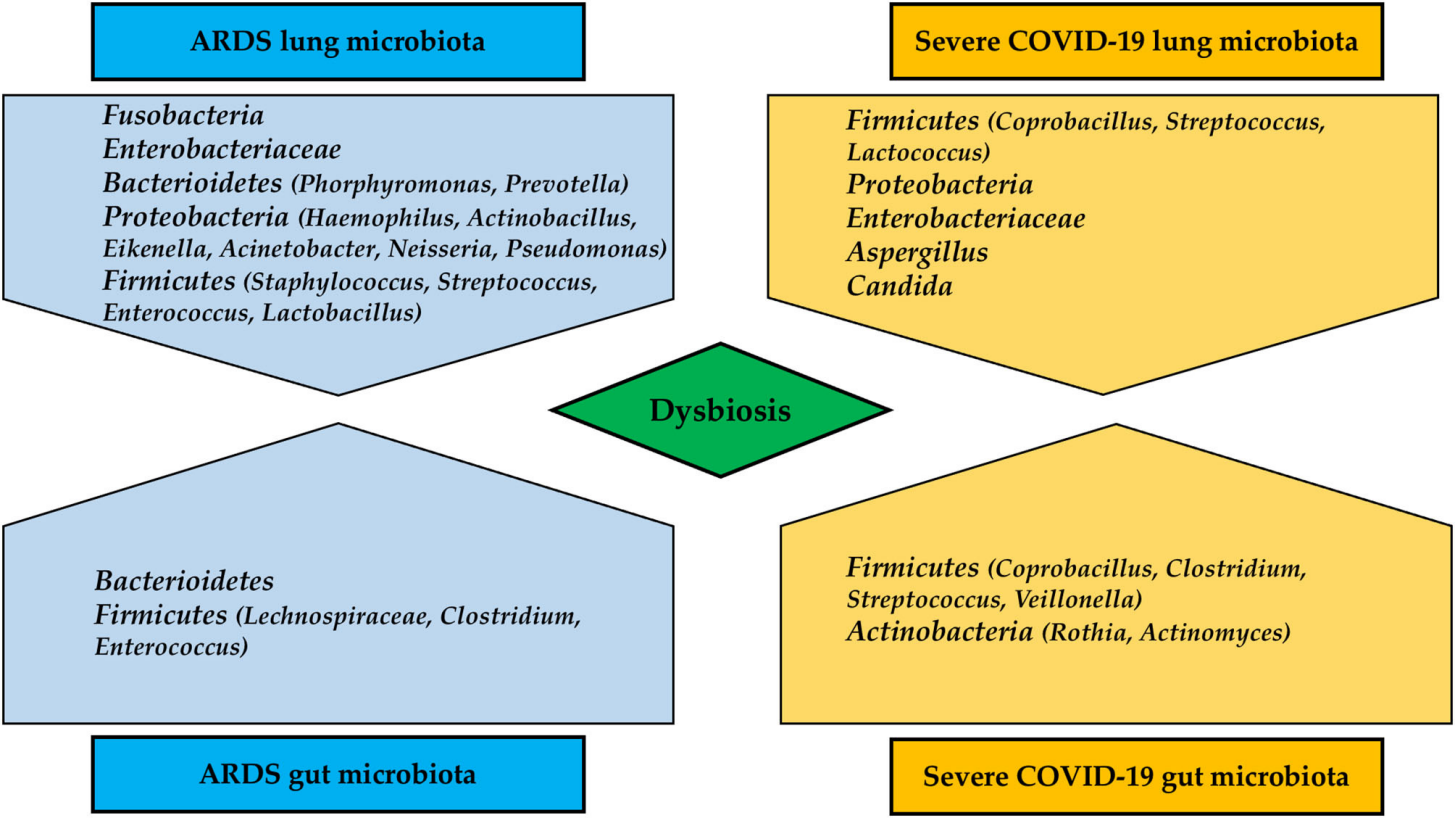
Differences in lung and gut microbiota composition in patients with severe COVID-19 pneumonia and typical ARDS.

The oral cavity is the second largest microbiota in humans, and *Neisseria, Corynebacterium, Leptotrichia, Streptococcus, Veillonella, Prevotella, Fusobacterium*, and *Capnocytophaga* are among the most common bacterial taxa (64). Similarly, the healthy lung microbiota is predominantly composed of *Streptococcus, Fusobacterium, Pseudomonas, Veillonella, Prevotella*, and *Capnocytophaga* (65). In a recent study of eight patients with SARS-CoV-2 infection, samples of bronchoalveolar lavage fluid (BALF) were sequenced for meta-transcriptome. The host variants varied from 0 to 51 due to a high rate of evolution of the virus. Differences in microbiota composition between healthy controls and those with SARS-CoV-2 were observed, although the variation was not specific (65). Opportunistic bacteria have been found in BALF of patients with COVID-19, particularly *Veillonella* and *Capnocytophaga*, which are commensal of the oral cavity (66). Another study on BALF of patients with COVID-19 revealed predominance of *Leptotrichia buccalis, Veillonella parvula, Capnocytophaga gingivalis*, and *Prevotella*, whereas *Acinetobacter baumannii, Klebsiella pneumoniae, Aspergillus flavus, Candida glabrata*, and *Candida albicans* were detected in nasal swabs (67, 68). Several studies demonstrated a higher incidence of ventilator-associated pneumonia (VAP) in patients with ARDS due to SARS-CoV-2 infection. In a multicentric study in 586 patients admitted to ICU, VAP incidence resulted as high as 29%, being *Pseudomonas aeruginosa* and *Staphylococcus aureus* the most involved microorganisms. Septic shock at VAP onset and ARDS were associated with fatality (69). Another study which compared 81 COVID-19 and 144 non-COVID-19 patients, concluded that those with SARS-CoV-2 infection were significantly more likely to develop VAP (Cox proportional hazard ratio 2.01, 95%CI 1.14–3.54, *p* = 0.0015). The organisms responsible of VAP and microbiome were similar between groups, but COVID-19 patients were more susceptible to *Aspergillus* and *Herpes* infections than general ICU patients (70). Again, data extracted by Rouzé et al. (71) from an European multicentric cohort of 1,576 patients concluded that lower respiratory tract infections associated to ventilation were significantly higher in patients with COVID-19 as compared to influenza patients, and those without viral infections. The most common causative pathogens included *Pseudomonas aeruginosa, Enterobacter spp*, and *Klebsiella spp*. Further studies are warranted to confirm the real incidence of lung dysbiosis and VAP in cases of severe COVID-19 pneumonia. Figure 2 depicts differences in lung and gut microbiota composition in patients with severe COVID-19 pneumonia and patients with typical ARDS.

## Risk Factors and Prevention of Dysbiosis in Severe COVID-19-ARDS

Recognizing the causes of dysbiosis in critical illness is challenging (16). Literature confirm that hospital admission may only partially alter patients' ecosystem, while increasing severity is commonly seen when implementing the level of cares. Indeed, several and impacting iatrogenic forces are applied during ICU care, thus affecting the physiology of the host, which in turn alters the community structures of resident microbes. In healthy or minimally ill individuals the elimination of pathogens is normally rapid and mediated by the passage through the intestinal tract via defecation. During critically illness, glucose and electrolyte disturbances, endogenous and exogenous opioids, sedatives and catecholamines, myorelaxants, poor oral hygiene, endotracheal or nasotracheal intubation, cuff pressure balance, body position, patients' transport and mobilization represent only few of the possible risk factors that may influence dysbiosis (72, 73). The consequent systemic response includes a lowering of the stomach and intestinal transit-time, drops in bile salt production, impairment of immunoglobulin type A production, and loss of the mucosal barrier. Moreover, the intestinal wall is often hypoperfused, leading to mucosal inflammation, altered oxygen gradient and increased nitrate concentration, while reducing the commensal bacteria in favor of the pathogens, and lowering the transit-time and pathogens' elimination. Additionally, when mechanical ventilation is applied, the ecological system of the lungs is highly stressed with possible impaired muco-ciliary clearance, depressed cough reflex and pathogens' overgrowth (16). If to these important grounds typical COVID-19 patients' comorbidities are added, the risk of dysbiosis is dramatically increased.

### Comorbidities of Patients With Severe COVID-19-ARDS and Their Role in Dysbiosis

The age of patients with severe COVID-19 is commonly high, and populations of gut bacteria normally change with age. In the gut of the elderly, less *Bifidobacteria* have been identified, maybe because of reduced gut epithelial barrier function, reduced immune function, and increased inflammation (74). In addition, obesity seems to be a typical characteristic of COVID-19, and it is associated with higher levels of pro-inflammatory cytokines and a poorer gut barrier. These mechanisms may favor the passage of gram-negative bacteria with possible endotoxemia (75). Intestinal bacteria along with their products (like SCFAs) play a key role in the protection of the mucosal intestinal barrier, and in the maintenance of adequate permeability through tight junctions, that may be down-regulated by pro-inflammatory cytokines and chemokines (76). Low-grade systemic inflammation is also present in those with chronic cardiovascular disease, type II diabetes mellitus, arthritis, and cancer. This may increase the risk of infection and altered microbiota (77). A great proportion of COVID-19 patients has hypertension (78). SCFAs has a crucial role in regulating blood pressure, while trimethyl amine-n-oxide (TMAO) is involved in atherosclerosis, hypertension, and coronary artery diseases' pathogenesis (79).

COVID-19 patients who present type II diabetes mellitus are around 30%. *Lactobacilli* are higher in diabetic patients, while the abundance of *Firmicutes* is correlated with inflammation (80). This basal diversity should be kept in mind when approaching dysbiosis in a COVID-19 patient with both SARS-CoV-2 infection and type II diabetes mellitus. Finally, according to the CDC's weekly report, around 35% of critically ill COVID-19 patients have an underlying chronic lung disease, such as asthma (81). As previously explained, the direct lung damage may be responsible for microbiota dysbiosis and over-infections. The airway mucosal barrier may lose the critical defense against SARS-CoV-2 and other infections (46).

### Indirect Risk Factors of Dysbiosis Associated With Mechanical Ventilation in COVID-19

#### Oral Hygiene and Aseptic Mouthwashes

Poor oral hygiene has been associated with increased incidence of pneumonia and dysbiosis in critically ills (82), and no scientific evidence exists yet to recommend mouthwashes to control the SARS-CoV-2 load in the oral cavity. Some antiseptic mouth rinses have antiviral ingredients able to decrease the viral load, but conclusive evidences are still limited. Besides, changes in the normal oropharyngeal flora as a consequence of poor oral hygiene could be related, not only to a greater ease of infection by SARS-CoV-2 with consequent higher viral load and greater severity (83), but also to secondary superinfections (84).

Common periodontal pathogens have been identified in the lungs of ICU patients, including *Treponema denticola, P. gingivalis, Fusobacterium nucleatum, Actinobacillus actinomycetemcomitans*, and *Veillonella parvula* (85, 86). In a cohort of 122,251 patients, the risk of pneumonia increased in those who did not engage in good oral care, including the presence of dental caries and missing teeth (87). The use of mouthwashes to prevent pneumonia is still debated. A randomized controlled trial (RCT) on 80 ICU patients who were randomized to receive Nanosil mouthwashes and chlorhexidine 0.12% for 5 days demonstrated that the pneumonia rate was reduced in the Nanosil group (2.7 vs. 23.7%, *p* = 0.008), but mortality was similar in both groups (88). Another trial investigating the role of peroxide hydrogen over normal saline in the prevention of pneumonia concluded that patients treated with peroxide hydrogen had a lower risk of contracting VAP (relative risk [RR], 2.6; 95% confidence interval [CI], 1.04–6.49, *p* = 0.0279) (89). Although no data on patients infected with SARS-CoV-2 are available, mouthwashes containing cetylpyridinium chloride reduced *in vitro* SARS-CoV-2 infectivity. The reduction of SARS-CoV-2 infectivity may reduce lung dysbiosis, but the novelty of this study is limited by the fact that, being *in vitro*, it cannot reproduce the real condition of an *in-vivo* oral flora (90). Moreover, the efficacy of mouthwashes with hydrogen peroxide has not been in doubt, especially their capacity to inactivate corona and influenza viruses (91, 92). In conclusion, irrespective of the mouthwash agent, maintaining good oral hygiene is an effective strategy to reduce the rate of over-infections in all ICU patients, especially in those with COVID-19 and ARDS which may present higher risk of superinfections (70, 71).

#### Endotracheal Intubation, Cuff Pressure Control, and Chest Physiotherapy

Unfortunately, no specific study in severe COVID-19 pneumonia is actually available and current suggestions come from ICU patients. The choice of nasal intubation over the endotracheal route should be weighed against several factors, including a higher level of comfort, less use of sedatives and analgesics, but also the higher incidence of sinusitis and possible translocation of nasal bacteria to the lungs (93). A study comparing patients intubated endotracheally with polyurethane tubes with continuous assessment of cuff pressure and subglottic drainage with patients intubated with PVC and intermittent cuff pressure measurements and intermittent subglottic drainage, demonstrated that prevention of VAP could be performed by using polyurethane tubes, performing continuous subglottic drainage, and continuous cuff pressure measurement (94). The use of chest physiotherapy maneuvers such as subglottic secretion drainage has been identified as a valuable adjuvant for the prevention of VAP in ICU patients. This technique is currently in use in several ICUs during the COVID-19 pandemic, although with limited resources and higher risks. A modified technique has recently been proposed by our group to limit airborne exposure (95). A recent meta-analysis investigated the real benefits of this maneuver, concluding that subglottic secretion drainage is effective but not significant in reducing VAP (RR, 0.56; 95%CI, 0.48–0.63, *p* = 0.841) (96). A recent RCT compared chest physiotherapy with controls for the prevention of VAP, and found that VAP occurred in 39% of the intervention group vs. 8% of the control group (odds ratio, 14; 95% CI, 0.03–0.56; *p* = 0.002); no differences were found in terms of mortality and length of ICU stay (97). However, a meta-analysis concluded that chest physiotherapy does not reduce the incidence of VAP, although these results should be viewed cautiously due to the heterogeneity of the studies and poor evidence (98).

#### Body Position

As understood by decades of research, body position plays a pivotal role in the development of pneumonia and lungs' dysbiosis (99). The lateral position is known to be effective for improving oxygenation in monolateral pneumonia (100), but severe COVID-19 pneumonia seems to interest both lungs (101). Besides, lateral position in COVID-19 is applied (102, 103). Despite the confirmed application of lateral position in COVID-19, its effects on possible superinfections and subsequent dysbiosis has not been investigated yet. The majority of literature concerning the effects of body position on superinfections and possible dysbiosis come from non-COVID-19 setting. A meta-analysis from 10 RCTs compared a semi-recumbent position (30°-60°) and a supine position (0°-10). The semi-recumbent position, with the higher elevation of the head of the bed, reduced the risk of VAP (14 vs. 40%; RR, 0.36; 95% CI, 0.25–0.5) (104). The lateral Trendelenburg position and a semi-recumbent position were compared in a recent RCT, which concluded that the semi-recumbent position was associated with a higher incidence of VAP than the lateral Trendelenburg (4 vs. 0.5%; RR, 0.13; 95% CI, 0.02–1.03; *p* = 0.04), with no differences in mortality and other secondary outcomes (105). Finally, the prone position, which assumes a pivotal role for severe COVID-19 with ARDS (101), did not seem to increase the incidence of VAP (incidence rate per 100 days of mechanical ventilation of 1.18 vs. 1.54 for supine and prone positions, respectively, *p* = 0.1) (106), as confirmed by a previous similar RCT (107). Similar results were obtained from a multicentric study on 586 COVID-19 patients (69). In conclusion, as stressed above, few studies investigating the role of body position on lung dysbiosis in severe COVID-19 pneumonia are currently available. We suggest that body position may play a role in the development of dysbiosis.

### Medications as Possible Risk Factors of Dysbiosis

#### Antibiotics

Numerous medications are administered in the ICU. Antibiotic consumption in ICUs is almost doubled that in non-ICU wards. During the COVID-19 pandemic, severely ill ICU patients received more antibiotics (66, 108). Antibiotic use is associated with important changes in gut microbial communities with a subsequent loss of the colonization resistance, a hallmark feature of the healthy gut microbiota, thus increasing the susceptibility to gastrointestinal infections by nosocomial pathogen (109, 110). Antibiotic exposure seems to increase the phyla of *Actinobacteria, Bacteroidetes, Firmicutes*, and *Proteobacteria* (111). The COVID-19 pandemic is associated with a higher and often unnecessary use of antibiotics in the early phases of the disease, in older people, and in mechanically ventilated patients (112, 113). Azithromycin is one of the largely used antibiotics in COVID-19 due to its antiviral and immunomodulatory effects *in vitro*, which include the interference with receptor mediated binding, viral lysosomal escape, intracellular pathways and enhancement of type I and III interferon expression (20). Besides, recent trials on the use of azithromycin combined or not with hydroxychloroquine in critically ill COVID-19 patients tended toward non-routine use (114). Similar results were obtained from a large observational study (115, 116). Another RCT on the use of azithromycin in hospitalized patients with COVID-19 is currently ongoing (117).

#### Sedatives, Analgesics, and Myorelaxants

Sedation and analgesia in mechanically ventilated COVID-19 patients are important pieces of this complex multisystemic puzzle. Patients with severe COVID-19 pneumonia and multiorgan disease are often kept sedated and curarized for longer periods than non-COVID-19 patients (median 10 days vs. 1 day) (118). Moreover, stopping sedatives, analgesics, and myorelaxants a greater proportion of COVID-19 patients experienced delirium (119). Growing evidence confirm the role of sedatives, analgesics opioids and myorelaxants on gut microbiota composition. Opioids receptors are located both in the digestive tract and central nervous system, and its effects on dysbiosis have been largely reported by literature. Moreover, some bacterial components can modify the expression of opioids receptors, changing the tolerance to pain (120). Larger studies are needed to confirm the effect of these medications on gut microbiota composition and outcome.

#### Inotropes and Vasopressors

Critically ill mechanically ventilated COVID-19 with ARDS frequently report the need of vasopressors and inotropes for treating septic shock or other multisystemic diseases (69, 121). Insights from animal models concluded that catecholamines may increase the growth of bacteria, virulence-associated factors, adhesions, and biofilm formation, while influencing the outcome of infections in many hosts (122). Inotropes have been associated with the growth of pathogens, and vasopressors inhibit growth (123, 124). To date, no evidence concerning the effects of inotropes and vasopressors on gut dysbiosis have been described.

#### Proton Pump Inhibitors (PPI) and H-2 Receptor Antagonists (H2RA)

PPI and H2RA are largely used in ICU for stress ulcer prophylaxis, and likely increases mortality but with low certainly evidence (125–127). The effect of PPI on gut microbiome has been largely investigated in animal studies. PPI showed increased intestinal permeability when compared to non-treated animals, thus changing the microbial composition, impairing colonization resistance, and inducing dysbiosis (128) and pneumonia in humans (129). This was also confirmed by other evidences in humans (130), but few specific investigations on COVID-19 are available yet. In a small monocentric study in 152 COVID-19 patients the impact of PPI was tested (131). Sixty-two patients were treated with PPI, of whom 48.4% without clear reason. Forty-eight percentage of patients treated with PPI, and only 20% of those non-treated presented with secondary infection. Forty-eight percentage of PPI treated patients and 12% of non-treated developed ARDS. The development of secondary infections remained significant after adjusting for other potential confounding (131). Although the sample size of this study is small, we believe that an association between the use of PPI and H2RA and superinfections in COVID-19 who are -per se- at higher risk of superinfections should be considered. Moreover, another study concluded that the pre-hospital use of PPI was associated with worse clinical outcome in hospitalized COVID-19 (132, 133).

#### Steroids

The Surviving Sepsis Campaign Coronavirus Disease 2019 recently published the first update of their known guidelines (134). High-quality evidence showing reduction in death and minimal adverse effects with short course of corticosteroids. Thus, the guidelines strongly recommended the use of a short course of systemic corticosteroids over not using corticosteroids. There are no trials comparing different corticosteroids with each other, but dexamethasone was associated with good treatment effect compared to no corticosteroids (135, 136). On one hand, corticosteroids reduce death and severity of COVID-19; on the other hand, corticosteroids remain mediators of the stress response that may enhance the hypothalamus-pituitary-adrenal axis which is implied in the control of immune response to stressor agents and intestinal microenvironment. Glucocorticoids may be therefore be involved in the alteration of gastrointestinal microbiota by enhancing the translocation of aerobe and gram negative enteric bacteria to extraintestinal tissues (137). A recent RCT reported no substantial differences in infections among critically ill COVID-19 patients treated with dexamethasone (21.9%) and those not treated (29.1%). However, few conclusive studies are warranted to confirm the real effects of corticosteroids on superinfections in severe COVID-19 pneumonia (138).

## Treatment Proposals

### Prebiotics and Probiotics

Probiotics are living micro-organisms which confer benefits to the host when administrated in adequate dose, and most used organisms include bifidobacteria, lactic acid bacteria, enterococci, and yeast (139). Probiotics usually have distinctive characteristics such as the ability of surviving under intestinal conditions, stimulating the immune system and acting against pathogens, also preventing health-care associated pneumonia (140). Furthermore, probiotics exert interesting properties by modulating cytokines production, interacting with TLRs, antagonizing pathogens in cell adhesion and mucin homeostasis, and by stimulating SCFAs production (141).

Probiotics act by enhancing epithelial barrier function and are anti-inflammatory, improving gut diversity and competing against opportunistic pathobionts for the same ecological niches in the gut (including competition for nutrients or cellular receptors on the mucosal surface). Specifically, they act by blocking or activating multiple signaling pathways (such as NF-kB and STAT1) and producing protective metabolites such as SCFAs. Gastrointestinal symptoms (including diarrhea) appear to be common in COVID-19, possibly reflecting alterations in the composition of gut microbiota (dysbiosis), inflammation and disruption of the epithelial barrier. In this context, administration of probiotics and/or prebiotics might be considered. As an example, Lactobacilli are well-known modulators of intestinal inflammation and immune response, so that their administration is recommended to counteract high level of inflammation, in prevention of diarrhea, and during infections sustained by enteric pathogens (139). Additionally, *Bifidobacteria*, are able to produce vitamins, enzymes, acetic and lactic acids, lowering the pH in the colon microenvironment and inhibiting (potential) pathogens (142). Evidence of beneficial effects, such as decreased infections frequency, shortening of the duration of episodes by 1–1.5 days, reduced shedding of rotaviruses or an increase in the production of rotavirus-specific antibodies, have been demonstrated for *Lactobacillus rhamnosus* GG (LGG), *L. casei* Shirota, *L. reuteri, Bifidobacterium animalis* ssp. lactis Bb-12, and a number of other probiotic strains (143).

Probiotics, prebiotics (formulation of nutrients exploited by probiotic bacteria), and symbiotics (a synergistic combinations of pro- and prebiotics) are currently used to improve gut dysbiosis, by favoring the proliferation of healthy protective bacteria in the intestine, ameliorating or preventing inflammation (balancing proinflammatory and immunoregulatory cytokines) and other intestinal diseases (144). The use of probiotics has also been associated with a reduction in the incidence and severity of VAP. Probiotics reduced the duration of mechanical ventilation in critically ill patients (145, 146). Specifically, use of the probiotic *Streptococcus salivarius* K12 has been proposed for patients with COVID-19 (146). Also, the presence of ACE2 was identified in certain probiotics strains. Oral delivery of human ACE2 through the probiotic species *Lactobacillus paracasei* increased ACE activity in the serum and tissues of mice. Similar results can be obtained with the bacteria-derived B38-CAP enzyme (147, 148). Recent research highlighted the role of mucin biopolymers as pivotal in regulating mucin production, which is implied in viral replication in the gut. Lactobacilli are known implementors of the mucus layer and glycocalyx, and inhibitors of pathogenic adherence, thus preventing intestinal inflammation (149). A recent network meta-analysis provided a rationale for the implementation of probiotics in preventing and treating COVID-19. They identified 90 genes potentially implicated in COVID-19 probiotics treatment. Moreover, the clearly shown that the application of probiotics could play a pivotal role on ACE2-mediated virus entry, activation of the systemic immune response, immunomodulatory pathways, lung tissue damage, cardiovascular complications, and altered metabolic pathways in the disease outcome (150). There are currently multiple lines of research with probiotics and numerous potential therapeutic indications, however studies with strong scientific evidence of therapeutic benefits are required.

### Fecal Microbiota Transplantation

Fecal microbiota transplantation (FMT) is gaining ground as a treatment option for certain changes in the gut microbiota. The mechanism of action of FMT requires a fecal suspension from a healthy donor deposited into the gastrointestinal tract of a patient by using an endoscope, nasal tube, or capsule. However, FMT is still considered “off label” except for recurrent or refractory *Clostridium difficile* infections, where reconstitution of the intestinal microbiota by FMT has proved extremely successful and has definitively confirmed the role of dysbiosis in the pathogenesis such infection. Only one study reported FMT in COVID-19 population (151). Because COVID-19 frequently presents with gastrointestinal symptoms (such as diarrhea), fecal transplantation could potentially contribute to spreading the virus. Therefore, the authors suggested careful identification of donors, considering typical symptoms and history of possible contacts, as well as donor testing for SARS-CoV-2 by real-time PCR (152). Eleven COVID-19 patients who received fecal microbiota transplantation resulted in altered peripheral lymphocyte subset, restored gut microbiota and alleviated gastrointestinal disorders (151). FMT efficacy may be affected by some microbial metabolites as primary bile acids (such as cholic acid and chenodeoxycholic acid), that are conjugated by the gut microbiota and bile salt hydrolase to form secondary bile acids (such as deoxycholic acid, lithocholic acid, and ursodeoxycholic acid) (153). The post-antibiotic expansion of *C. difficile* population was shown to be strongly associated with the depletion of secondary bile salts, consequently to an antibiotic-mediated depletion of microbial taxa mediating the conversion of primary into secondary bile acids (154). Primary and secondary bile acids may also exert anti-inflammatory properties and inhibit several viruses by modulating the cytokine-storm via NF-kB (influenza A, and other viruses) (155).

Most intriguingly, while the treatment's success of FMT mostly revolves around intestinal viable healthy bacteria that are transferred through fecal suspensions, it should be considered that the viable bacteria fraction may not be the only factor affecting the recipient's biology. Viruses, archaea, fungi, donor's colonocytes, immunoglobulin, protists, and a number of metabolites, made by the donor's commensal bacteria (as SCFAs) or intestinal cells, can be implanted, potentially triggering a plethora of functionally different effects (156).

### Dietary Composition

In the acute phase of ICU admission, inflammation, energy expenditure, and catabolic metabolism are enhanced (157). During their stay in the ICU, patients often develop post-ventilation-acquired dysphagia and ICU-acquired weakness, which mean nutritional support has a pivotal role in maintaining the necessary muscular strength to help wean patients from the ventilator (158–160). Moreover, critical illness is considered to be a major environmental factor in influencing gut homeostasis and dysbiosis, and nutritional therapy could play an essential role in these processes. Among the various environmental factors, indeed, diet is a source of dominant variation of the whole gut microbial community (161). As an example, nutritional models based on plant-based foods were shown to promote a more favorable gut microbiota profile based on the high amount of dietary fiber and SCFA (162). Therefore, nutrition may exert different indirect effect on intestinal function by modulating the gut microbial composition. A recent study on fecal samples of patients with COVID-19 revealed a high abundance of bacterial species *Collinsella aerofaciens, Collinsella tanakaei, Streptococcus infantis, Morganella morganii*, and higher nucleotide *de novo* biosynthesis, amino acid biosynthesis, and glycolysis. These were distinct from fecal samples of patients without COVID-19 who had higher abundance of SCFAs-producing bacteria, including *Parabacteroides merdae, Bacteroides stercoris, Alistipes onderdonkii*, and *Lachnospiraceae bacterium 1_1_57FAA* (163). It is now well-recognized that SCFAs exert several beneficial effects, influencing a number metabolic (as the lipids, cholesterol and glucose metabolism) and inflammatory (as the butyrate-mediated inhibition of macrophagic NF-κB or inhibition of the LPS-induced cytokines IL-6 and IL-12p40) responses (164).

Colon bacteria respond to fermentable substrates provided by the diet to produce SCFAs and gases through anaerobic metabolism (165). Within this context, dietary intake is an essential factor for resilience of patients' gut microbiota and its impact on upper respiratory tract infections (145).

The use of early enteral nutrition has been associated with improved immunologic function, decreased bacterial translocation, and better mucosal integrity (111). Moreover, the composition of enteral nutrition has a great impact on intestinal homeostasis. The gut microbiota is normally preserved be feeding with various dietary components in different concentrations. With that in mind, an inadequate dietary composition may alter the composition of the intestinal microbiota, thus increasing the growth of opportunistic pathogens over commensals (111).

General nutritional consideration for ICU patients should be applied to COVID-19 patients (166). As COVID-19 is frequently associated with gastrointestinal symptoms, patients can be at high risk of refeeding syndrome. If this risk is present, SCCM/ASPEN guidelines recommend starting at ~25% of the target energy intake (whether enteral or parenteral) (166). The ESPEN guideline estimates around 27 kcal/kg body weight/day, based on total energy expenditure, for patients aged >65 years with multiple comorbidities, or 30 kcal/kg body weight/day, based on total energy expenditure, for severely underweight patients with multiple comorbidities and older adults (individually adjusted on the basis of nutritional status, physical activity, and disease status) (167). An energy goal of 15–20 kcal/kg actual body weight should be reached within the first week of nutritional support even in COVID-19 (166). Recently, in a prospective observational study Cereda et al. in mechanically ventilated COVID-19 patients who have been fed with a low caloric intake in the early phase of ICU admission, found a higher risk of death. Additionally, patients with mild obesity were associated with higher mortality, while those with moderate-severe obesity were more difficult to wean from the ventilator (168). Early overfeeding should be avoided, because aggressive caloric intake can cause hyperglycemia or the need for insulin therapy (169). In case of contraindications to oral and enteral nutrition, parenteral nutrition should be implemented and increased within 3–7 days (170, 171). In critically ill patients intolerant to enteral feeding, intravenous erythromycin should be considered as the first-line prokinetic therapy, followed by intravenous metoclopramide or a combination of both (170). Prone positioning is being used with increasing frequency to treat both typical ARDS and respiratory distress in severe COVID-19 pneumonia. Traditionally, this leads to forced periods of rest from enteral nutrition (172), although enteral nutrition has recently been demonstrated to be feasible and safe in the prone position as well (173). In patients at high risk of aspiration, post-pyloric enteral nutrition can be provided instead (170) to reduce the possible risks related to prone positioning and development of pneumonia.

The specific recommendations for nutritional management in COVID-19 (167) suggest that a high-energy, low-to-normal carbohydrate (based on diabetic status and glycemic control), normal-to-high protein diet should be considered. Contrasting findings are available concerning the optimal protein intake for critically ill patients (174). Protein intake can influence the catabolic response. During the catabolic phase, within the first 10 days of ICU admission, a reduction in muscle mass of up to 1 kg/day in patients with multiorgan dysfunction can occur (175). A recent RCT compared enteral feeding with high-intact-protein formula (VHPF) with a standard high-protein formula (SHPF). The VHPF facilitated feeding without increasing energy intake, which is consistent with previous ESPEN guidelines (176). However, early high protein intake is associated with a lower mortality rate only in patients with a low skeletal muscle area at hospital admission, not in those with a normal skeletal muscle area (177). Another study found that improvement in daily protein intake could reduce 3-month mortality after hospital discharge (178). A standard high-protein (>20%) isosmotic enteral formula may be used in the early phase of critical illness, with possible addition of fibers (if tolerated) for maintenance of gut microbiota function (166). Consider 1 g protein/kg body weight/day in older persons (individually adjusted on the basis of nutritional status, physical activity, disease status, and tolerance), and 1.2–2.0 g protein/kg body weight/day (166) in patients with multiple comorbidities (167). An isocaloric, high-protein diet is recommended for obese patients, especially guided by urinary nitrogen losses (170); if this measurement is not available, a protein intake of 1.3 g/kg should be considered. The latest ESPEN guidelines recommend a daily protein intake of 1.3 g/kg, delivered progressively (170). However, a great number of COVID-19 patients require continuous renal replacement therapy as part of the systemic multiorgan dysfunction that they manifest. Thus, specific consideration of protein intake during the use of such filters for renal depurations should have been counted by novel guidelines (179).

**Figure 3:**
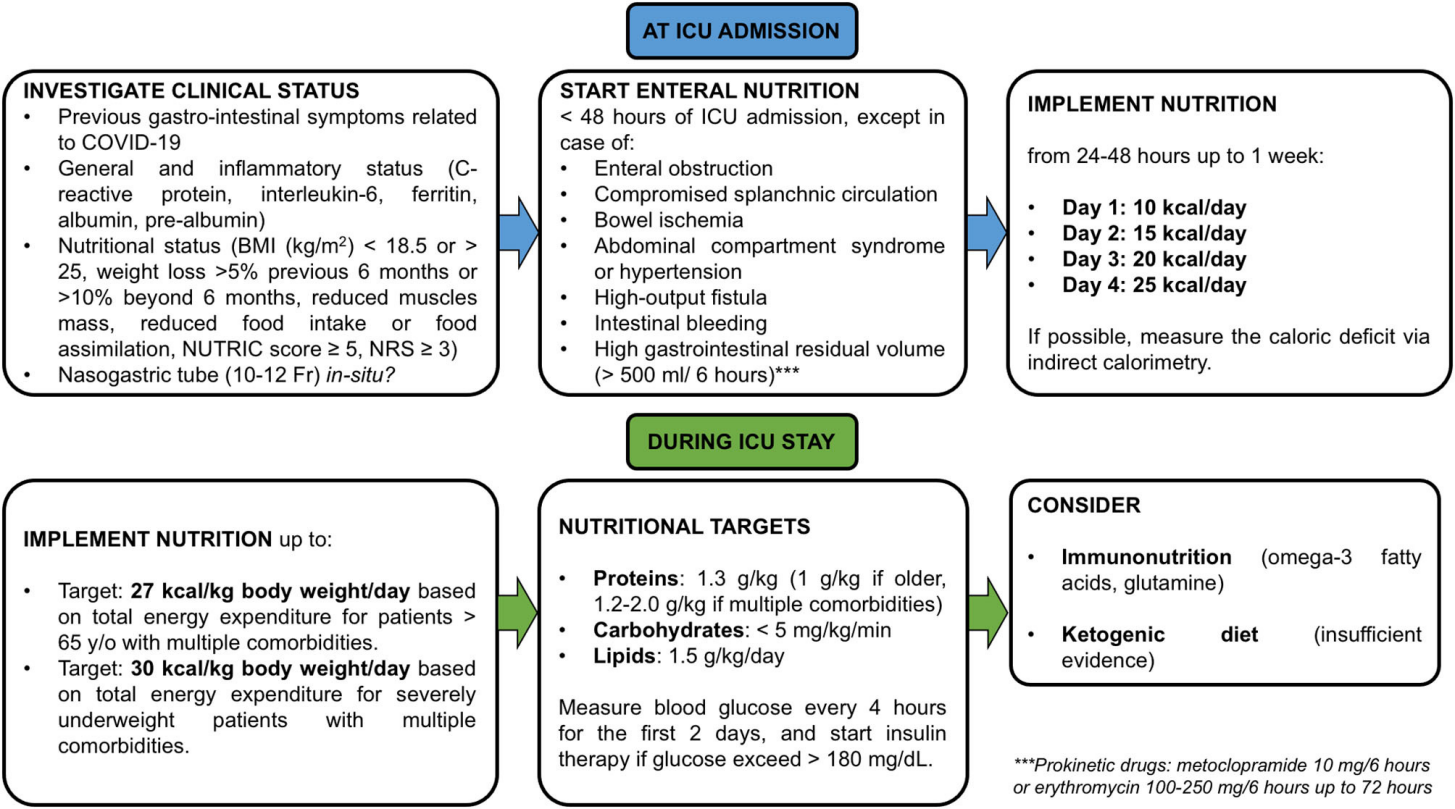
Dietetic recommendations in cases of COVID-19 with ARDS. Nutritional recommendations for critically ill patients with COVID-19 and ARDS from ICU admission to ICU stay. NRS, nutritional risk screening. Each nutritional support is suggested to be calculated on Ideal body weight.

The amount of glucose, whether parenteral or via carbohydrates by enteral feeding, should not exceed 5 mg/kg/min (170). However, current guidelines in COVID-19 did not account that hypertension, obesity, and diabetes mellitus are the most prevalent comorbidities that may alter patients' metabolic profile (180). Similar consideration may be done for lipids administration in a population composed by a large number of obese patients, as COVID-19 population is (180). Indeed, guidelines recommend that intravenous lipids for parenteral nutrition should not exceed 1.5 g/kg/day (170). The intake of carbohydrates and fat should be adapted according to energy ratio of 50:50 from fat and carbohydrates for ventilated patients (167). Additionally, a ketogenic diet for obese or diabetic patients should be considered (181). Since COVID-19 often leads to liver and renal failure, parenteral Gln dipeptide should not be administered (170). Blood glucose should be measured at ICU admission and at least every 4 h for the first 2 days. Insulin therapy should begin when glucose levels exceed 180 mg/dL (170). Triglyceride levels should be considered in cases of prolonged sedation with propofol or prolonged administration of intravenous lipid emulsion for parenteral nutrition (166). Adequate intake of vitamins and minerals is paramount for the prevention of viral infections. Particularly, vitamins A, E, B6, B12, C, and D; zinc; selenium; iron; and omega-3 polyunsaturated fatty acids should be administered with a view to ameliorating clinical outcomes, as advised for other viral illnesses (167, 182). A recent RCT demonstrated that no mortality advantages were found in critically ill patients who received early implementation of vitamin D (183). In COVID-19, a serum 25-hydroxyvitamin-D level of around 30 mg/mL reduced the risk for adverse clinical outcomes (184), but further studies are needed to confirm these findings. General dietetic recommendations for critically ill patients with COVID-19-ARDS are depicted in Figure 3.

### Other Nutritional Interventions to Modulate Dysbiosis in COVID-19

Other nutritional interventions have been proposed to modulate the cytokine storm in ARDS and COVID-19, but studies are lacking. Therefore, the following sentences represent an overview of nutritional treatments for immune and inflammatory dysfunctions in COVID-19 patients. ARDS is considered an overwhelming systemic inflammatory process. Patients with COVID-19 frequently present with hypoalbuminemia and lymphopenia, which may reflect malnutrition and hyperinflammation and have been associated with a negative prognosis (1). Although the albumin level should not be considered a nutritional marker in patients with active inflammation, prealbumin levels are associated with progression to ARDS (185). Patients who survived severe COVID-19 pneumonia often present significant functional limitations, and experience higher morbidity and mortality (186).

#### Immunonutrition

Immunonutrition has been proposed for patients with severe COVID-19 pneumonia (187, 188) because supplementary immunonutrients and antioxidants have been shown to promote favorable outcomes in the general critically ill population (186, 189, 190). The severity of disease influences the efficacy of immunonutrition (190). In one meta-analysis, immunonutrition reduced mortality and improved oxygenation in patients with ARDS (191); however, more recent studies failed to replicate these findings (186, 192–194). Several products are available to provide immunonutrition. Broadly, these consist of antioxidant vitamins (e.g., vitamin E, vitamin C, carotene), trace elements (e.g., selenium, zinc), essential amino acids (e.g., glutamine, arginine), and essential fatty acids (e.g., omega-3 fatty acids, eicosapentaenoic acid, docosahexaenoic acid, linolenic acid) (186).

Monounsaturated and polyunsaturated fatty acids are involved in cytokine production (190). When immunonutritional enrichment of fatty acids is administered, many components of the immune response are modulated and suppressed (190) by modification of the lipid bilayer of multiple cell types. Omega-3 fatty acids are essential lipids that are able to suppress pro-inflammatory eicosanoid biosynthesis, reduce lung permeability, inhibit inflammation by enhancing T cell function, and decrease pulmonary edema (189, 195). On the other hand, the administration of omega-6 fatty acids may have opposite effects. Thus, the intravenous administration of lipid-enriched solutions may be detrimental, increasing mortality and complications in critically ill patients, because of the infusion of high amounts of omega-6 fatty acids (196).

**Figure 4:**
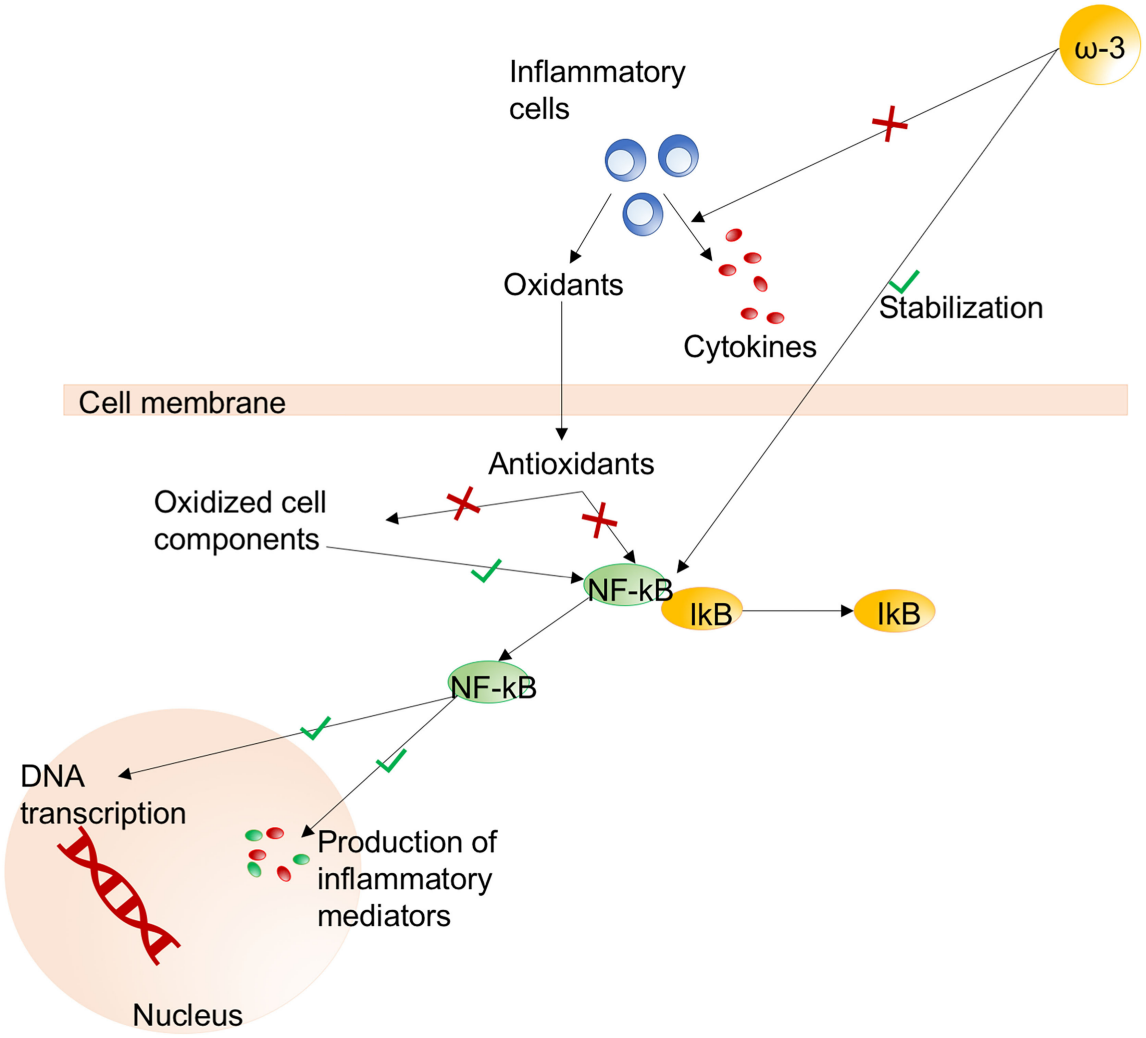
Immunonutrition. The main pathways activated (green) or inhibited (red) during immunonutritional therapy. Effects of omega-3 fatty acids on stabilization of the NF-κB/IκB pathway and reduced production of cytokines from inflammatory cells. Effects of NF-κB on the nucleus include DNA transcription and production of inflammatory mediators. NF-κB, nuclear factor kappa-B; ω-3, omega-3; DNA, deoxyribonucleic acid. Modified from Grimble (100).

Glutamine and arginine are sulfur-containing amino acids that have been proposed as components of immunonutrition for their immunomodulatory properties. Particularly, the properties of glutamine include improvement of gut barrier function and immunomodulation of lymphocyte, neutrophil, and macrophage function (186). Glutamine also enhances glutathione synthesis and cell proliferation, thus enhancing antioxidant mechanisms. Likewise, arginine enhances nitric oxide synthesis, lymphocyte function, growth hormone production, and anabolism (189). Arginine is synthesized from proline and participates in the synthesis of ornithine, which is essential for immune function. Arginine deficiency has been found to correlate with suppression of T cell proliferation and cluster of differentiation (CD)3 (190). An RCT of a specific anti-inflammatory and antioxidant nutritional therapy regimen for patients with COVID-19 is ongoing (197). Precursors of cysteine may be administered exogenously in the form of N-acetylcysteine or procysteine, although cysteine and methionine are not easily captured into cells (190). The putative mechanisms of immunonutrition are summarized in Figure 4. Although glutamine and antioxidants could be considered in patients with oxidative stress, benefits to outcomes have not been reported. On the contrary, an increase in mortality was found in critically ill patients with multiorgan failure (198). Therefore, caution is needed since conclusive evidences are not available yet.

#### Ketogenic Diet

Ketogenic diet is a nutritional alternative to mitigate inflammation in COVID-19 patients. The ketogenic diet is a low-carbohydrate, high-fat nutritional support strategy that promotes metabolic ketosis. This has proved to be efficient in controlling glucose levels and body weight, and in promoting anti-inflammatory effects in obesity and type 2 diabetes (199, 200). Ketogenic diets were initially proposed to control refractory status epilepticus and protect the central nervous system (201, 202). Over time, evidence emerged that very low-carbohydrate diets decreased energy intake while improving lipid and glucose homeostasis (203), as well as decreasing levels of inflammatory markers (181). Preliminary results in a murine model of beta coronavirus infection demonstrated that ketones protect against systemic inflammatory response (204). The rationale for using ketogenic diet in COVID-19 is summarized in the following paragraphs. Furthermore, a trial investigating the use of ketogenic diet for patients with COVID-19 is ongoing (205).

**Figure 5:**
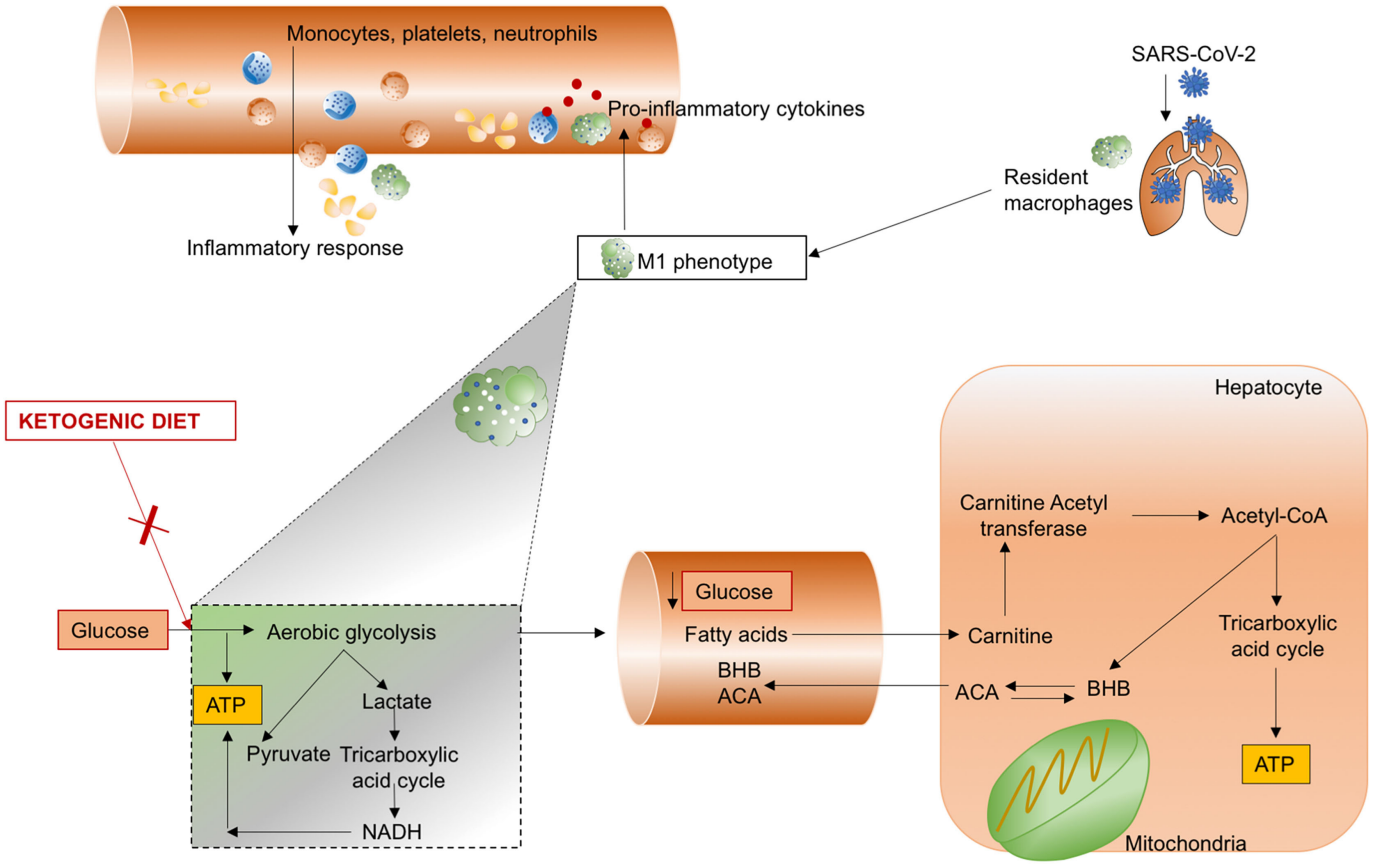
Ketogenic diet. SARS-CoV-2 infects the lung and induces hyperinflammation with recall of monocytes, platelets, and neutrophils by macrophages polarized to the M1 phenotype. A ketogenic diet is able to reduce the synthesis of adenosine triphosphate (ATP) from glucose by limiting aerobic glycolysis, usually implicated in the production of lactate and pyruvate, and activation of the tricarboxylic acid cycle, culminating in increased nicotinamide adenine dinucleotide (NADH). Glucose concentration in the blood is reduced, thereby increasing the production of β-hydroxybutyrate (BHB) and acetoacetate (ACA) from hepatocyte mitochondria.

The release of inflammatory cytokines and caspase-1, as occurs in SARS-CoV-2 infection following the activation of innate immunity in response to damage-associated molecular pattern (DAMPs) (7), can be modulated by the nod-like receptor protein-3 (NLRP3) inflammasome (206). Ablation of NLRP3 is able to attenuate type 2 diabetes and atherosclerosis (7), which have been identified in most patients with severe COVID-19. During a ketogenic diet, alternative sources of energy are produced by the liver, including the ketone bodies β-hydroxybutyrate (BHB) and acetoacetate (ACA), to maintain the metabolic functions of the brain, heart, and skeletal muscles. The increased consumption of liver glycogen stores that is characteristic of all ketogenic diets is also associated with altered immune cell function. Specifically, the use of lactate as a source of mitochondrial oxidative energy plays a key role in the production of innate immune type I cells and interferon type I, which are effective in the host defense against viral infections (207). In an experimental mouse model, caloric restriction implemented through a ketogenic diet was found to exert anti-inflammatory effects; ketone bodies attenuated caspase-1 activation and IL-1β secretion by modulating the NLRP3 inflammasome (208). Recent research has proposed that the inhibitor of glycolysis, deoxy-D-glucose, could be a reasonable therapeutic strategy for SARS-CoV-2 infection (209), because it has been found to reduce the duration of ventilator support and partial pressure of carbon dioxide in patients with acute respiratory failure (210). The mechanisms of action of a ketogenic diet are summarized in Figure 5.

## Conclusions

Dysbiotic states of the microbiota may impact on the pathogenesis, as well as on the complexity, of immune and inflammatory diseases. Several mechanisms have been identified as potential targets to reduce inflammation and secondary infections. Particularly, novel nutritional interventions have been proposed to regulate the mechanisms underlying dysbiosis of the lung and intestinal microbiota. However, further studies on patients with severe COVID-19 are needed to confirm the effective benefit of such interventions.

## Author Contributions

DB: conceptualization, writing original draft, review, and editing. CR, AF, ST, SS, VD, DG, AV, MB, IB, NP, LB, and AT: review and editing. PR and PP: writing, review, and editing. All authors contributed to the article and approved the submitted version.

## Conflict of Interest

The authors declare that the research was conducted in the absence of any commercial or financial relationships that could be construed as a potential conflict of interest.
